# Inconsistencies in the published rabbit ribosomal rRNAs: a proposal for uniformity in sequence and site numbering

**DOI:** 10.1261/rna.080294.124

**Published:** 2025-06

**Authors:** Swastik De, Michelle Zhou, Zuben P. Brown, Raymond N. Burton-Smith, Yaser Hashem, Tatyana V. Pestova, Christopher U.T. Hellen, Joachim Frank

**Affiliations:** 1 Department of Biochemistry and Molecular Biophysics, Columbia University, New York, New York 10032, USA; 2 Irvington High School, Irvington, New York 10533, USA; 3 Exploratory Research Center on Life and Living Systems (ExCELLS), National Institutes of Natural Sciences, Okazaki, Aichi 444-8787, Japan; 4 Institut Européen de Chimie et Biologie, U1212 Inserm, Université de Bordeaux, 33607 Pessac, France; 5 Department of Cell Biology, SUNY Downstate Medical Center, Brooklyn, New York 11203, USA; 6 Department of Biological Sciences, Columbia University, New York, New York 10027, USA

**Keywords:** ribosome, 28S rRNA, 18S rRNA, 5S rRNA, 5.8S rRNA, and cryo-electron microscopy

## Abstract

Examination of all publicly available *Oryctolagus cuniculus* (rabbit) ribosome cryo-EM structures reveals numerous confusing inconsistencies. First, there are a plethora of single-nucleotide differences among the various rabbit 28S and 18S rRNA structures. Second, two nucleotides are absent from the NCBI Reference Sequence for the 18S rRNA gene. Moving forward, we propose using the Broad Institute's rabbit whole-genome shotgun sequence and numbering to reduce modeling ambiguity and improve consistency between ribosome models.

## INTRODUCTION

The translation of stored genetic information into proteins is central to all domains of life. The eukaryotic ribosome, a complex biomolecular machine composed of the small (40S) and large (60S) subunits, facilitates the reading of sequential codons in messenger RNA (mRNA) by transfer RNA (tRNA), converting this information into the language of amino acids and functional proteins. Both subunits contain multiple riboproteins bound to a backbone of different-length RNA strands: the 28S, 5.8S, and 5S rRNAs for the mammalian 60S subunit and the 18S rRNA for the 40S subunit. In recent years, while the majority of structural data for the eukaryotic ribosome has been acquired through cryo-electron microscopy (cryo-EM) and increasingly cryo-electron tomography (cryo-ET), some structures of yeast and human ribosomes have also been determined using X-ray crystallography. The data predominantly come from four species: two yeast species (*Saccharomyces cerevisiae* and *Kluyveromyces lactis*, with 262 and 14 structures, respectively), human (244 structures), and rabbit (121 structures), with all other eukaryotic organisms accounting for only 194 models (Table 1, as of 09/08/24). While the structure of the human ribosome is of high interest for understanding human biology and disease mechanisms, rabbit ribosomes are often used in their place because they can be purified in large quantities from rabbit reticulocyte lysate (RRL)—the most widely used system for in vitro analysis of translation (Jackson and Hunt 1983) and used for reconstitution of ribosomal complexes (e.g., Hashem et al. 2013; Hilal et al. 2022). RRL is also used to prepare programmed translation reactions that are arrested at specific stages, which can then be purified for cryo-EM analysis (e.g., Bhatt et al. 2021; Shao et al. 2016; Simonetti et al. 2016, 2020).

**TABLE 1.** Presently available structures of eukaryotic ribosomes (as of September 8, 2024, prepared using RADtool [Hassan et al. 2023])

	80S structures	60S structures	40S structures
Eukarya	377	274	184
*Saccharomyces cerevisiae*	155	87	20
*Kluyveromyces lactis*	2	11	1
*Oryctolagus cuniculus*	64	21	36
*Sus scrofa*	11	7	3
*Homo sapiens*	80	89	75
*Mus musculus*	4	1	4
*Plasmodium falciparum*	4	2	2
*Canis lupus familiaris*	1	0	0
*Triticum aestivum*	5	1	2
*Drosophila melanogaster*	4	0	0
*Spinacia oleracea*	4	4	2
*Vairimorpha necatrix*	1	0	0
*Arabidopsis thaliana*	1	0	0
*Neurospora crassa*	4	2	1
*Tetrahymena thermophila*	1	4	6
*Paranosema locustae*	1	0	0
*Thermochaetoides thermophila*	5	19	0
*Danio rerio*	2	0	0
*Xenopus laevis*	1	0	0
*Candida albicans*	8	0	0
*Spraguea lophi*	4	0	0
*Giardia* spp.	7	2	3
*Encephalitozoon cuniculi*	1	0	0
*Rattus norvegicus*	1	0	0

Structural investigations and extensive biochemical characterization have significantly advanced our understanding of eukaryotic ribosomal biology, providing insights into ribosome assembly (Baßler and Hurt 2019; Broeck and Klinge 2024), modification (Sloan et al. 2017), initiation (Jackson et al. 2010; Hashem and Frank 2018; Querido et al. 2024), elongation (Dever et al. 2018; Djumagulov et al. 2021; Holm et al. 2023; Milicevic et al. 2024), termination (Hellen 2018), quality control (D'Orazio and Green 2021), and overall structure (Yusupova and Yusupov 2014, 2017).

In this report, we investigated errors present in the rabbit ribosomal rRNA sequences within published PDB models. We also examined how these errors have affected the current structures and have prepared sequence-corrected structures for rabbit 28S, 18S, 5.8S, and 5S rRNAs.

### Discrepancies among published 28S rRNA sequences

The 60S ribosomal subunit is composed of 5S, 5.8S, and 28S rRNAs, along with various ribosomal proteins. The 28S rRNA functions as a ribozyme, catalyzing peptide bond formation essential for protein synthesis (Yusupova and Yusupov 2014). This catalytic property is associated with RNA's ability to fold into compact structures, creating cavities that serve as binding sites for ligands. Furthermore, studies have shown that specific elements of the 28S rRNA, such as es27l and es39l, play critical roles in binding, recruiting, coordinating, and regulating ribosome-associated protein complexes like the nascent polypeptide-associated complex (NAC) (Knorr et al. 2019; Lentzsch et al. 2024). Inconsistencies among published 28S rRNA *Oryctolagus cuniculus* sequences create issues for comparative studies and may lead to inaccurate conclusions. Aligning these sequences to a consistent reference would minimize ambiguities, improve research accuracy, and support the development of new therapies, especially for diseases like Alzheimer's (Payão et al. 1998). Since the rabbit ribosome is a commonly used model, having accurate and widely accepted numbering is crucial for pinpointing and understanding rRNA modifications, which are significant in the context of certain cancers. For example, human ribosome modifications have been precisely mapped by multiple groups, highlighting the importance of standardized references in ribosomal research (Krogh et al. 2020; Temaj et al. 2022; Barozzi et al. 2023; Cui et al. 2024; Holvec et al. 2024).

We compared rabbit 28S rRNA sequences from various sources, including published PDB structures and template sequences from NCBI GenBank, RNA Central, ENA, and Rfam databases, to identify the discrepancies and evaluate their impact. Seven high-resolution PDB structures from different groups published as rabbit ribosomes were selected to avoid any lab-specific biases.

To identify a reliable sequence as our template, we conducted an extensive search through several sequence databases, including NCBI GenBank, NCBI RefSeq, RNA Central, Rfam, NIH Biosample, NIH Bioproject, and the European Nucleotide Archive (ENA). Initially, we suspected that the absence of a dependable 28S rRNA sequence for rabbits might have contributed to numerous sequence errors in the PDB structures. However, we identified a reliable source in GenBank entry AAGW00000000.2, which corresponds to the *O. cuniculus* (Thorbecke inbred breed) whole-genome sequencing project, released in 2005 and re-curated in August 2009. This GenBank entry is linked to multiple ENA, Rfam, and RNA Central entries, all curated as the 28S rRNA sequence of *O. cuniculus*. The template sequence used for our study is found in RNA Central under accession code URS00009AB771_9986. This sequence originated from the Broad Institute's rabbit whole-genome sequencing trials (OryCun2.0), which employed shotgun sequencing methods to assemble the entire rabbit genome. A team of Broad Institute scientists completed a deep coverage (7×) draft of the rabbit genome, offering significantly higher accuracy compared to previous sequences. We also considered the NCBI Genome assembly UM_NZW_1.0, which is a New Zealand White rabbit sequence from the Shanghai Institutes for Biological Sciences (Bai et al. 2021). Although the New Zealand White sequence offers higher quality with 40× redundant coverage compared to the 7× coverage of OryCun2.0, we opted for the OryCun2.0 sequence for several reasons: (1) it is more thoroughly curated (providing the complete length of over 5000 nt for 28S sequence compared to the ∼3600 nt of the UM_NZW_1.0 sequence), and (2) it is more commonly used by the biochemical and structural groups. The availability of this high-quality, thoroughly curated 28S rRNA sequence from OryCun2.0 provided a reliable template for our comparative analysis, which was essential for accurately identifying and understanding discrepancies observed in rabbit 28S rRNA sequences reported in various PDB structures.

Using the Broad Institute's rabbit sequence as a template for alignment, we compared the seven selected PDB entries with 28S rRNAs with the template's sequence. These comparisons revealed numerous discrepancies, ranging from single-nucleotide changes to substantial insertions and deletions, indicating a lack of consistency and accuracy in reported structures. In this report, we have documented single-nucleotide differences to highlight the issues arising from the lack of a validated consensus sequence. While major insertions and deletions could be tabulated, we deliberately chose not to focus on these variations due to various complications associated with them. Instead, we emphasized on the need for a standardized consensus sequence, which is crucial for accurate structural data interpretation across rRNA variants. Without such a sequence, alignment discrepancies are inevitable, and each variant would have a unique and inconsistent numbering system beyond the first indel. We chose to omit a detailed discussion of major indels to maintain clarity and focus on the necessity of a unified reference framework.

The number of single-nucleotide changes varies from none to as many as 47 (PDB 6P5I) (Acosta-Reyes et al. 2019). The extent of these variabilities suggests that the inconsistencies are caused by a combination of factors, such as using different methodologies, starting models, and sequence correction practices.

The origins of the starting models play a crucial role in determining the final sequence accuracy. For instance, PDB 6GZ5 from the Spahn group matches the template rabbit sequence, showing no nucleotide changes. This high level of sequence accuracy may be attributed to a careful sequence correction process conducted by the authors, who thoughtfully edited the starting human ribosome model (PDB 5AJO) to align with the rabbit sequence (Behrmann et al. 2015). This approach underscores the importance of thorough sequence verification and correction, especially when working with models from closely related species. In contrast, the 6P5I PDB entry from our own laboratory (Acosta-Reyes et al. 2019) exhibited several single-nucleotide mutations, deletions, and insertions, likely due to the initial use of a *K. lactis* ribosome structure (PDB 5IT9/5IT7) as a starting point (Murray et al. 2016). This example highlights the potential challenges that can arise when using models from more distantly related species without comprehensive sequence validation.

**FIGURE 1.**
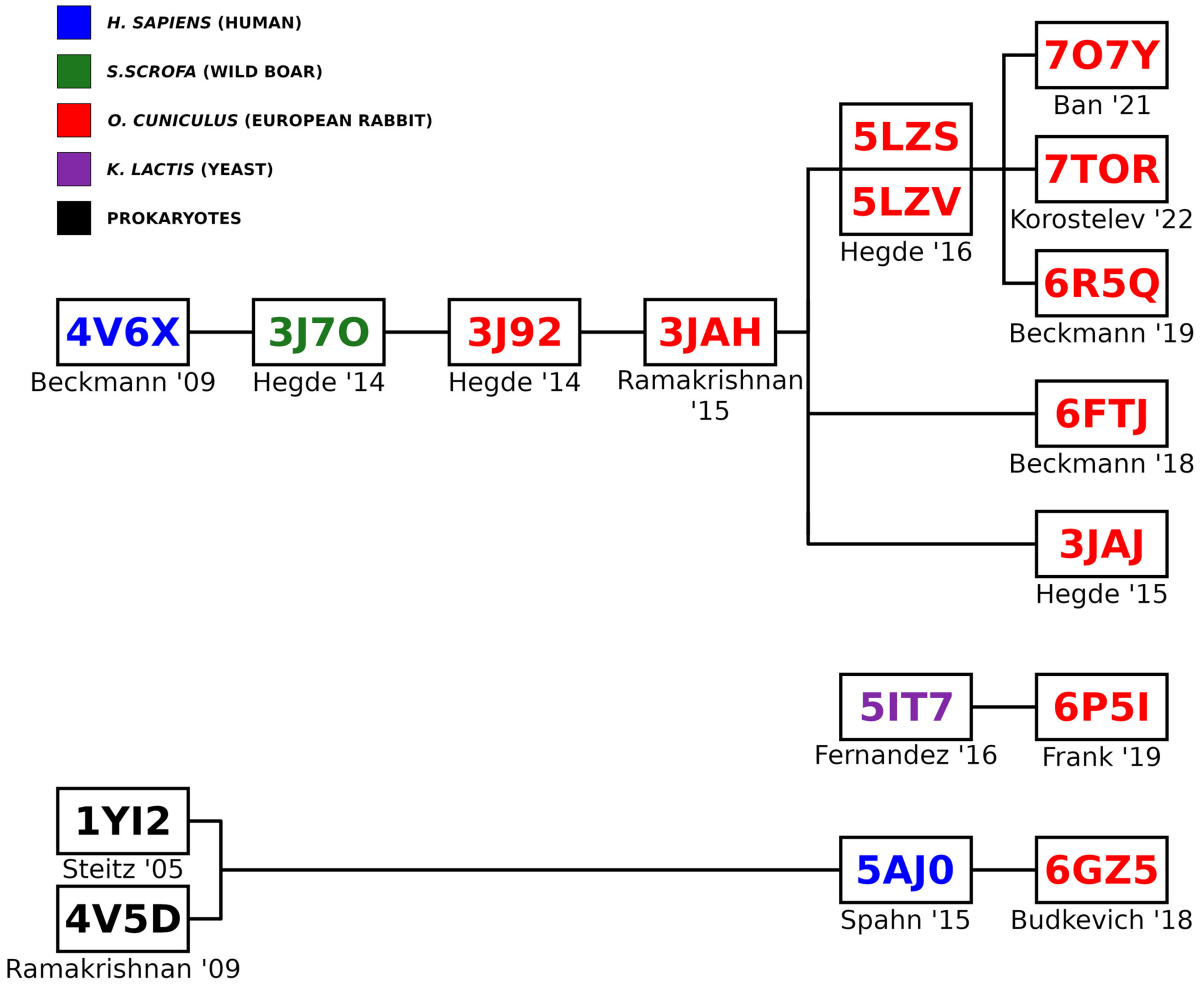
Origins of rabbit 80S ribosome Protein Data Bank entries. The PDB entries were sourced from the following studies: 4V6X (Anger et al. 2013), 3J7O (Voorhees et al. 2014), 3J92 (Shao et al. 2015), 3JAH (Brown et al. 2015), 5LZS (Shao et al. 2016), 5LZV (Shao et al. 2016), 7O7Y (Bhatt et al. 2021), 7TOR (Loveland et al. 2022), 6R5Q (Shanmuganathan et al. 2019), 6FTJ (Braunger et al. 2018), 3JAJ (Voorhees and Hegde 2015), 5IT7 (Murray et al. 2016), 6P5I (Acosta-Reyes et al. 2019), 1YI2 (Tu et al. 2005), 4V5D (Voorhees et al. 2009), 5AJO (Behrmann et al. 2015), and 6GZ5 (Flis et al. 2018).

Interestingly, many of the PDB entries we analyzed trace their origins back to a common progenitor PDB entry, 3JAH, a structure of a rabbit ribosome published by the Ramakrishnan Lab (Brown et al. 2015). This entry was itself derived from another rabbit ribosome model, PDB 3J92 (Shao et al. 2015), which in turn was based on an earlier boar ribosome model, PDB 3J7O (Voorhees et al. 2014). Ultimately, these sequences can be traced back to a single human ribosome model, 4V6X, published by the Beckmann group (Fig. 1; Anger et al. 2013). This lineage illustrates how a single starting model can influence subsequent studies, and it suggests that any inaccuracies or assumptions in earlier models may persist through multiple iterations. Shao et al. (2016) utilized the OryCun2.0 genome sequence, complemented by direct sequencing from rabbit ribosomes (RRL), to refine and correct the sequences in high-confidence regions of the 28S (bases 725–965, 1271–2888, and 3584–3867).

**FIGURE 2.**
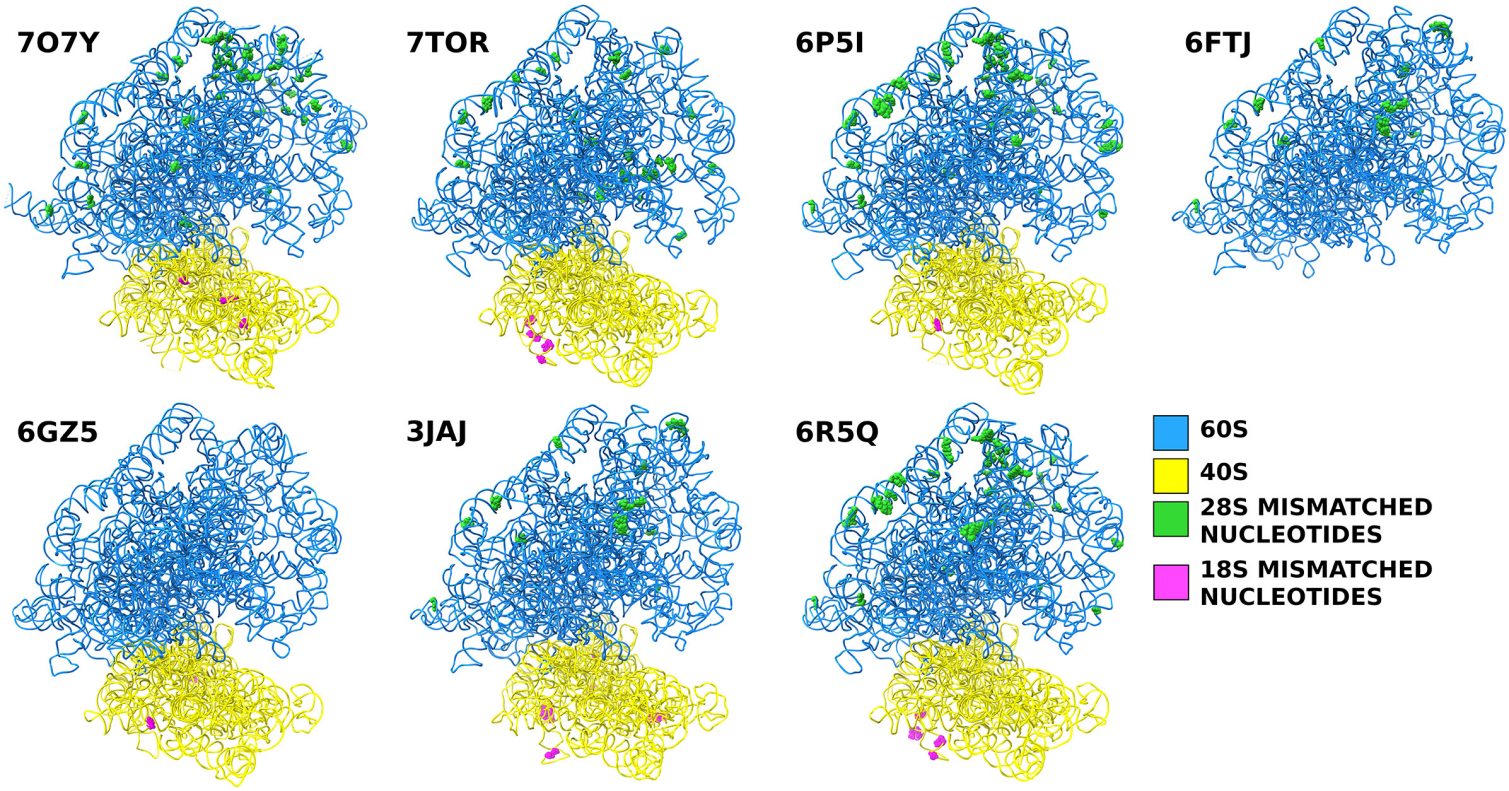
Positions of the single-nucleotide mismatches in the selected PDB entries compared to the rabbit template sequence.

**FIGURE 3.**
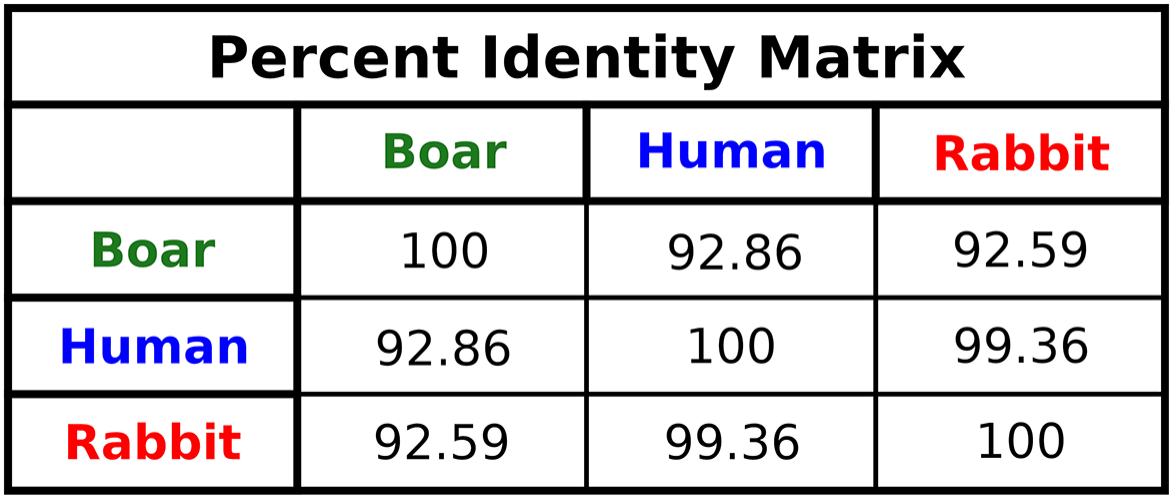
Percentage identities between the boar (URS0000C12F10_9823), human (URS00027DEF72_9606), and rabbit (URS00009AB771_9986) 28S rRNA sequences.

Our analysis also found that some PDB entries labeled as rabbit contained sequences from other species, such as humans or boars. For example, the 28S rRNA sequence of PDB 3J92, categorized as rabbit, was derived from a boar PDB (3J7O) without any nucleotide changes (Figs. 1, 2; Voorhees et al. 2014; Shao et al. 2015). While boar and rabbit 28S RNA Central template sequences are 92.59% identical (Fig. 3), this observation raises important questions about the accuracy and validity of species-specific annotations in PDB entries and the conclusions drawn from such data. Additionally, a comparison between the RefSeq 28S *H. sapiens* rRNA (NR_003287) used to build the 28S rRNA of PDB entry 4V6X and RNA Central's rabbit template sequence (URS00009AB771_9986) revealed a surprisingly low percent identity of 84.91%. This finding highlights the significant sequence divergence that can exist even among related mammalian species, emphasizing the necessity for precise sequence verification across all organisms.

### Discrepancies among published 18S rRNA sequences

During all stages of ribosome function, the aminoacyl (A), peptidyl (P), and exit (E) sites on the 40S subunit are essential for the precise translation of mRNA. These sites are composed of both ribosomal protein residues and nucleotides from the 18S rRNA. Specific nucleotides of the 18S rRNA (as detailed in Table 2) are crucial for positioning tRNA, ensuring accurate codon-anticodon base-pairing, interacting with initiation factors, and forming binding sites for viral IRES sequences (Pisarev et al. 2006; Quade et al. 2015; Yamamoto et al. 2015; Simonetti et al. 2016; Abaeva et al. 2020). For instance, the Kozak interaction highlights the significance of the +4 nt interacting with A1819 (or A1825) of the 18S rRNA (Pisarev et al. 2006; Simonetti et al. 2020). The positioning of tRNA involves C1331 for the A-site tRNA and C1701/U1248 for the P-site tRNA in humans (Anger et al. 2013), with interactions of DHX29 with helices h16, h17, and h34 of 18S rRNA, the Sec-insertion domain of SBP2 binding to h33 of 18S rRNA, tRNA ASL with G1575 and A1576, mRNA with G1150, and additional interactions described by Petrychenko et al. for initiator tRNA with nt 1248 and 1701, and mRNA with nt 626, 961, 1207, and 1701 (Hussain et al. 2014; Simonetti et al. 2020; Sweeney et al. 2021; Hilal et al. 2022; Petrychenko et al. 2024) and engage with sense or stop codons (Brown et al. 2015; Matheisl et al. 2015). Furthermore, 18S rRNA nucleotides are crucial for binding and stabilizing IRES structures, such as U1115 and C1116 of ES7 in 18S rRNA interacting with A136 and G266 of the HCV IRES (Quade et al. 2015; Yamamoto et al. 2015; Brown et al. 2022), while C1701 and U1830 of 18S rRNA bind A1058 and U1248 of the HalV IRES (Abaeva et al. 2020). Additional interactions involving A1824-A1825/G626 of 18S rRNA and A3760/A4255 of 28S rRNA with the Israeli Acute Paralysis Virus IRES (Acosta-Reyes et al. 2019) have also been reported.

**TABLE 2.** Features of the ribosomal 18S rRNA for the most commonly available models in the Protein Data Bank

	Note	*H. sapiens*	*O. cuniculus*		*S. cerevisiae*	*K. lactis*
			2009 shotgun	1982 NCBI reference		
Sequence ID		NR_145820.1	NW_003159740.1	NR_033238.1	NG_063315.1	NG_063256.1
Length		1869	1869	1863	1730	1748
A-site (G626)	A1825 contact	626	626	636	577	576
A-site (C1331)	tRNA base	1331	1331	1327	1274	1273
A-site (A1825)	mRNA contact	1825	1825	1819	1756	1754
P-site (U1248)	tRNA base	1248	1248	1244	1191	1190
P-site (C1701)	tRNA base	1701	1701	1696	1637	1635

In terms of the sequence length in modeled human or yeast ribosomes, the 18S rRNA appears to be consistent, while in rabbit ribosomes, the 18S rRNA length is variable, either matching the human 18S rRNA (GenBank accession no. NR_145820.1; 1869 nt; 45 models), the 1982 NCBI Reference Sequence for rabbit 18S rRNA (GenBank accession no. NR_033238.1; 1863 nt; seven models), or—in the case of the current highest-resolution rabbit ribosomal structure available—the human sequence, though out of register by +1 (1870 nt; four models). While slightly different sequences do not impact the biological interpretation of these ribosome complexes, they do introduce ambiguity in analysis across papers as identical features are referred to using different identifiers (see Table 2).

To determine the correct 18S rRNA sequence, we examined all publicly available rabbit ribosome structures determined using cryo-EM at better than 4.0 Å resolution. While doing so, we noticed that the 1982 NCBI Reference Sequence for the rabbit 18S rRNA is partially incorrect (see Table 2 for the complete list). All cryo-EM density maps reveal at least 2 nt missing from the 1982 NCBI Reference Sequence that are present in the human sequence (G183 and C1513). Although the nucleotide sequence of helix 18 (h18) is not entirely conserved across all species, it contains seven sequence clusters, sometimes referred to as h18 universals (Noller et al. 2022). Helix 18 has an inversion of bases in the 1982 NCBI rabbit 18S rRNA sequence. The bases G613 and C614 are reportedly reversed for the rabbit sequence (Connaughton et al. 1984; Rairkar et al. 1988), but examination of high-resolution ribosome maps (e.g., Bhatt et al. 2021) shows apparent density supporting the GC ordering seen in humans and other eukaryotic species. Together, this suggests that the sequence identity and length of the 1982 NCBI Reference Sequence for the rabbit 18S rRNA—which has been used for some cryo-EM models, biochemical experiments on rabbit ribosomes, and primer design—is partially incorrect (e.g., Pisarev et al. 2006; Simonetti et al. 2020).

The Broad Institute's whole-genome shotgun sequencing of *O. cuniculus* (OryCun2.0) reported a sequence that shares 99.5% identity with the human 18S, having only 2 nt gaps. This shotgun sequence matches the human 18S sequence in length (1869 nt), includes the conserved GC sequence in h18, and has both G183 and C1513 that are visibly present in all rabbit cryo-EM maps.

We used the Broad Institute's *O. cuniculus* Thorbecke inbred whole-genome shotgun sequence to analyze rabbit 18S rRNA sequences (OryCun2.0). This template sequence provides a highly accurate baseline for comparing the 18S rRNA sequences reported in various PDB structures. The 18S template sequence we used is found in RNA Central under the accession code URS00006F07B6_9986.

To systematically evaluate these discrepancies, we aligned six 18S rRNA sequences from published PDBs with the Broad Institute's rabbit sequence as a reference template. This comparative analysis exposed a range of inaccuracies, including single-nucleotide substitutions and substantial insertions and deletions. For this report, we have concentrated on single-nucleotide variations, setting aside issues related to insertions and deletions. This decision was driven by the realization that addressing these insertions and deletions would compromise structural integrity and make accurately remodeling the affected segments extremely challenging, even with the presently available structural information.

The sequences from these PDB entries are predominantly consistent with the reference template. For instance, the 18S rRNA sequence from PDB entry 3JAJ is 99.37% identical to the reference template, with eleven single-nucleotide differences observed.

Similarly, PDB entry 6GZ5 shows an even higher level of sequence identity, at 99.82%, with only two single-nucleotide variations from the template. In contrast, PDB entry 6P5I aligns with the template at 99.63%, revealing nine single-nucleotide changes. PDB entry 6R5Q presents a sequence identity of 99.41%, with six discrepancies. Furthermore, PDB entry 7O7Y exhibits a 99.84% identity with just 3 nt differences. Lastly, PDB entry 7TOR shows a sequence identity of 99.41%, with six single-nucleotide differences from the template. While relatively minor, these variations are noteworthy and underscore the necessity for precision in structural biology studies.

Overall, the different PDB entries have very high sequence identities, ranging from 99.37% to 99.84%. Although there are only a few discrepancies, we must note that high accuracy is critical for precise structural interpretations and functional analyses, as minor differences can have significant implications for understanding the structure and function of rRNA within the ribosome.

This study emphasizes the importance of using accurate reference sequences for comparative analysis in structural biology. The minimal variations observed across these PDB entries suggest that the 40S structures are generally well-aligned with the reference template. However, careful attention to the remaining discrepancies is essential for ensuring the accuracy of ribosome models and advancing our knowledge of ribosomal mechanisms and functions.

### Discrepancies among published 5.8S and 5S sequences

We also used 5.8S (URS00006CE1FB_9986) and 5S (URS00006C8ED4_9986) template sequences from RNA Central to conduct a similar analysis. The 5.8S sequences in the published PDB entries matched the template sequence, with only a few indels at both ends. In contrast, the 5S rRNA showed several single-nucleotide variations as well as indel discrepancies. Notably, the 5S template sequence consists of 144 nt, while the PDB entries contain only 119 nt.

### Conclusion

The discrepancies we have found have profound implications for structural biology. Inaccurate and inconsistent rRNA sequences in published PDB entries can lead to misinterpretations of ribosome structure and function, potentially affecting the validity of many downstream research findings and applications. The propagation of incorrect sequences through successive studies exacerbates these issues, leading to a cascade of errors and misannotations. To address these challenges, we propose that all future rabbit ribosome models use the numbering and sequence from the Broad Institute's rabbit genome sequencing project.

Recent studies highlight the functional role of rDNA sequence and epigenetic variability across biological processes. Variant rRNA alleles show tissue-specific expression and respond to environmental stresses like early-life nutrition, influencing phenotypes such as memory and cancer (Holland et al. 2016; Parks et al. 2018; Milenkovic et al. 2025). Genetic variation between individual rDNA units can influence transcriptional output, potentially leading to the production of variant ribosomes (Rodriguez-Algarra et al. 2022), which in turn could lead to preferential translation of specific mRNAs, as suggested by the ribosome filter hypothesis (Mauro and Edelman 2002). Developmentally, different rRNA variants are required for embryogenesis in mice (Tseng et al. 2008). In bacteria, divergent rRNAs reprogram ribosomes for stress adaptation to environmental changes (Song et al. 2019). These findings reveal the significance of rDNA variability in shaping gene expression and phenotypic outcomes. Although rabbits similarly possess multiple cistrons for their rRNAs just like many other organisms, potentially introducing sequence variability (Martin-DeLeon 1980; Hori et al. 2023; Rothschild et al. 2024), our study provides a crucial foundation for understanding ribosome structure and function by establishing a consensus sequence.

In this way, we believe our study enables more accurate and meaningful interpretations of structural results, ultimately advancing our understanding of ribosome biology. We have also deposited the rRNA sequence-corrected model into the model archive (ma-u3zgf) to facilitate the use of correct sequences by the field. This will also maintain consistency in numbering important ribosomal subunit sites between rabbit and human ribosomes. By adopting rigorous sequence verification and correction practices and utilizing trusted template sequences, we can ensure more accurate and reliable models, advancing our understanding of ribosome structure.

## MATERIALS AND METHODS

For this study, we selected seven PDB entries (7O7Y, 7TOR, 6P5I, 6FTJ, 6GZ5, 3JAJ, and 6R5Q) from seven different published studies. We utilized the curated RNA Central entry URS00009AB771_9986 as a consensus sequence for the rabbit 28S rRNA template, which is accessible under GenBank accession AAGW00000000.2, Rfam accession RF02543, and ENA accession GL019111 (Broad Institute's rabbit whole-genome sequencing trials [OryCun2.0]). The 28S rRNA sequences from each PDB were downloaded from RCSB and aligned against the consensus sequence using Clustal Omega (EMBL-EBI) (Sievers et al. 2011). This alignment was reviewed for single-nucleotide discrepancies, which were meticulously recorded. Insertions and deletions were excluded from this analysis. The 18S, 5S, and 5.8S rRNA sequences were also downloaded and aligned against the consensus 18S (URS00006F07B6_9986), 5S (URS00006C8ED4_9986), and 5.8S (URS00006CE1FB_9986) rRNA sequences. We used ChimeraX (Meng et al. 2023) to examine structural details, verify alignment fidelity, and resolve any alignment issues. Additionally, Coot (Emsley and Cowtan 2004) was used to address and correct sequence errors as they arose during analysis. Finally, each refined structure was further refined against our unpublished *O. cuniculus* 80S ribosome maps and already published *O. cuniculus* 40S maps (Brown et al. 2022) to ensure rabbit-specific ribosomal conformation using phenix.refine (Afonine et al. 2012). This step provided an additional level of precision to our analysis.

## DATA DEPOSITION

Starting from the atomic model of the rabbit 80S ribosome (PDB ID 6GZ5), we corrected the rRNA sequences based on the OryCun2.0 sequence and deposited the revised model in the Model Archive, accessible under the identifier ma-u3zgf.

## Figures and Tables

**Zuben P. Brown:**
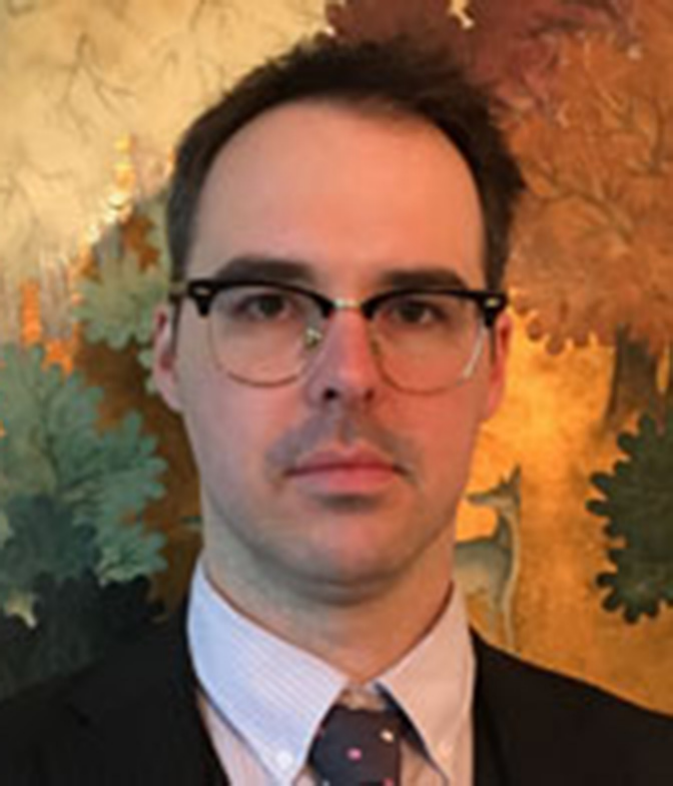


**Swastik De:**
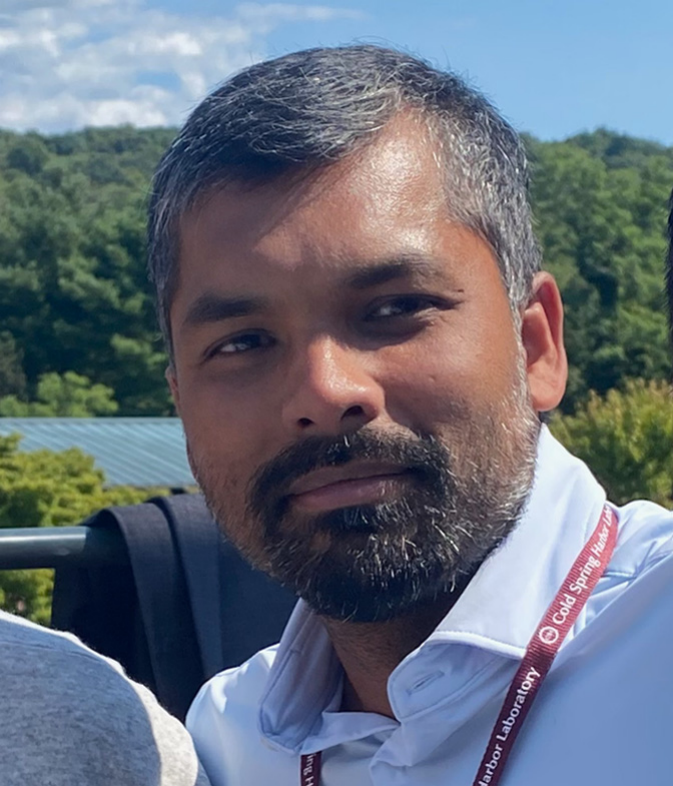


**Michelle Zhou:**
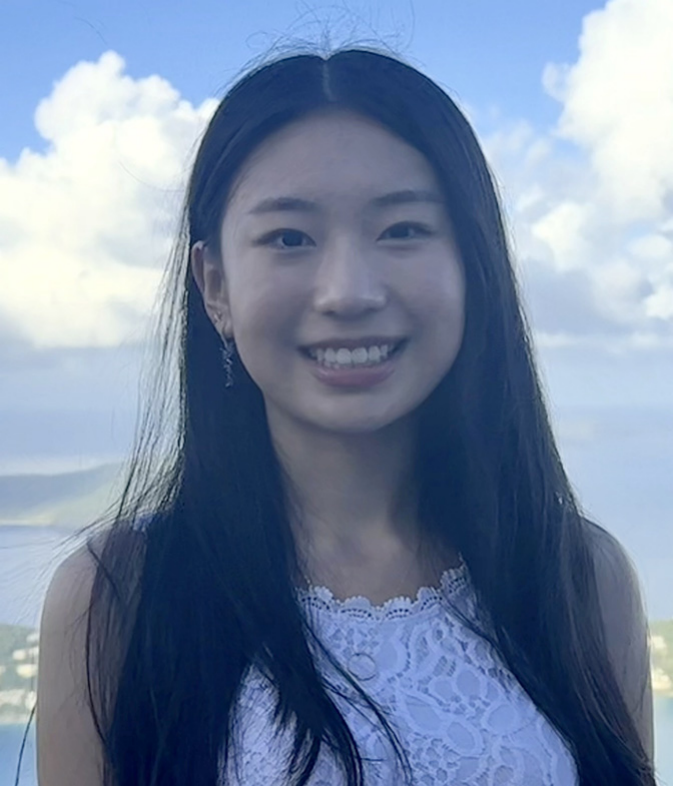


## MEET THE FIRST AUTHORS

***Meet the First Author(s)* is an editorial feature within *RNA*, in which the first author(s) of research-based papers in each issue have the opportunity to introduce themselves and their work to readers of *RNA* and the RNA research community. Swastik De, Michelle Zhou, and Zuben P. Brown are co-first authors of this paper, “Inconsistencies in the published rabbit ribosomal rRNAs: a proposal for uniformity in sequence and site numbering.” Swastik is a postdoctoral researcher in the Frank Lab at Columbia University Irving Medical Center (CUIMC). Michelle is a senior at Irvington High School in New York, who has trained and participated in research in the Frank Lab at CUIMC, focusing on RNA and ribosome initiation factors. Zuben, formerly a postdoctoral researcher in the Frank Lab at CUIMC, is now a Senior Account Manager of the Cryo-EM unit at Thermo Fisher Scientific.**

**What are the major results described in your paper and how do they impact this branch of the field?**

Based on our analysis of published sequences showing inconsistencies, we recommend using the Broad Institute's Rabbit Genome Project (OryCun2.0) rRNA as the consensus sequence for future studies on *Oryctolagus cuniculus* 28S rRNA. Currently published PDB sequences are inconsistent, leading to potential misinterpretations of ribosome structure and function. Our study provides a more reliable reference, improving the accuracy of structural analyses and advancing our understanding of ribosome biology.

**What led you to study RNA or this aspect of RNA science?**

**MZ**: Nucleic acids are vital carriers of biological information, with RNA playing a key role in understanding living systems, human health, and disease. My interests lie in synthetic bioengineering and the development of drugs and proteins—areas where RNA research is essential for optimizing biological systems.

**SD and ZPB**: Ribosomes are fundamental to cellular life, serving as the molecular machines responsible for protein synthesis. At the Frank Lab, we investigate how ribosomes function following precise regulatory mechanisms critical for cell survival. Our research focuses on uncovering the structural and functional dynamics that drive ribosomal activity.

**During the course of these experiments, were there any surprising results or particular difficulties that altered your thinking and subsequent focus?**

We investigated the origins of rRNA sequences used for creating various models of ribosomes and were surprised to find significant discrepancies. Some sequences labeled as rabbit rRNA actually originated from a boar model, others were derived from human sequences, and two were traced back to bacterial sources. In particular, these findings reveal that inaccuracies in *O. cuniculus* rRNA sequences have been perpetuated across multiple models, leading to a cascade of possible errors and misannotations in ribosomal research.

**What are your subsequent near- or long-term career plans?**

**SD:** I'm currently a a postdoc at the Frank Lab and will be applying for academic positions.

**MZ:** I'm applying to colleges and plan to major in bioengineering. I intend to continue conducting biochemistry and cell biology research in college, and I hope to attend medical school after finishing my undergraduate studies.

**ZPB:** I'm currently a Senior Account Manager for Cryo-EM at Thermo Fisher Scientific.

**What were the strongest aspects of your collaboration as co-first authors?**

Our collaboration was effective because we leveraged each other's expertise. Swastik concentrated on the 28S sequences, Michelle handled the overall curation and sequence alignments, and Zuben focused on the 18S sequences, ensuring comprehensive coverage of the project. We maintained open lines of communication, frequently troubleshooting challenges together and seamlessly integrating our findings. By clearly dividing tasks while staying aligned on the broader vision, we maximized productivity without redundancy. This collaborative approach not only enhanced the quality of our research but also made the experience deeply rewarding.

**How did you decide to work together as co-first authors?**

This project began as a 40S sequence correction when Zuben, then in the Frank Lab, was studying IRES-mediated translation initiation in the rabbit ribosome in collaboration with the Hellen Lab. Around the same time, Swastik identified similar numbering inconsistencies in the 60S subunit he was working on. After Zuben left for his current position, Professor Joachim Frank asked Swastik to integrate both projects. During her internship in the Frank Lab, Michelle assisted Swastik in tabulating and tracking these inconsistencies.
